# Methods for Antifungal Susceptibility Testing of the *Cryptococcus neoformans*/*C. gattii* Complex: Strengths and Limitations

**DOI:** 10.3390/jof9050542

**Published:** 2023-05-05

**Authors:** Ana Espinel-Ingroff, Emilia Cantón

**Affiliations:** 1 VCU Medical Center, Richmond, VA 23298, USA; 2 Severe Infection Research Group, Health Research Institute Hospital La Fe, 46026 Valencia, Spain

**Keywords:** detection resistance, cryptococcal isolates, ECVs, mutant detection, *Cryptococcus* isolates, cryptococcal species/genotypes

## Abstract

When method-dependent categorical endpoints are available, namely either BPs or ECVs, MICs could aid in selecting the best treatment agent(s). BPs can categorize an isolate as either susceptible or resistant while the ECVs/ECOFFs can distinguish the wild type (WT, no known resistance mechanisms) from the Non-WT (NWT, harboring resistant mechanisms). Our literature review focused on the *Cryptococcus* species complex (SC) and the available methods and categorization endpoints. We also covered the incidence of these infections as well as the numerous *Cryptococcus neoformans* SC and *C. gattii* SC genotypes. The most important agents to treat cryptococcal infections are fluconazole (widely used), amphotericin B, and flucytosine. We provide data from the collaborative study that defined CLSI fluconazole ECVs for the most common cryptococcal species or genotypes and modes. EUCAST ECVs/ECOFFs are not yet available for fluconazole. We have summarized the incidence of cryptococccal infections (2000–2015) where fluconazole MICs were obtained by reference and commercial antifungal susceptibility tests. This occurrence is documented all over the world and those fluconazole MICs are mostly categorized by available CLSI ECVs/BPs as “resistant” instead of non-susceptible strains, including those by the commercial methods. As expected, the agreement between the CLSI and commercial methods is variable because SYO and Etest data could yield low/variable agreement (<90%) versus the CLSI method. Therefore, since BPs/ECVs are species and method dependent, why not gather sufficient MICs by commercial methods and define the required ECVs for these species?

## 1. Introduction

### 1.1. Background and Epidemiology

The incidence of cryptococcal infections is difficult to calculate. Most infections occur among HIV/AIDS patients (0.4 to 1.3 cases per 100,000 population) with a mortality rate of about 12% [1]. The estimated incidence of cryptococcal meningitis occurring worldwide is 152,000/year; most of these cases are reported in sub-Saharan Africa [1,2]. www.cdc.gov/fungal/diseases/cryptococcosis-neoformans/statistics (accessed on 20 March 2023). The epidemiology of the *Cryptococcus* species complex (SC) is well known and briefly summarized below. Among these species, most clinical isolates are *C. neoformans*; *C. gattii* has been isolated in the U.S., mostly in the Pacific Northwest area [2]. Considering that recent research has discovered the complex genetic composition of this group, genotyping is recommended. The *C. gattii* genotype distribution is region dependent and this species is more frequently isolated from infections among AIDS patients [2,3]. By 2011, phylogenetic analysis and genotyping studies clarified the diversity among the *C. gattii*/*C. neoformans* (SC) as follows [3,4,5,6]: *C. neoformans* var. *grubii* and *C. neoformans* var. *neoformans* are two distinctive species and five species are found within the *C. gattii* SC. In a more recent report, the incidence among 233 globally collected isolates of these species was as follows: *C. neoformans*/VNI, as expected, was the most prevalent genotype followed by *C. neoformans*/VNII (34 strains, 14.6%), *C. deneoformans*/VNIV (24 strains, 10.3%), *C. bacillisporus*/VGIII (17 strains, 7.3%), *C. gattii*/VGI (6 strains, 2.6%), *C. neoformans* × *deneoformans* hybrid/VNIII (5 strains, 2.1%), and *C. deuterogattii*/VGII (1 strain, 0.4%) [7].

Some of these facts were also summarized in another study with a collection of 5686 Cryptococcal isolates from clinical, environmental, and veterinary strains as reported by the Latin American Cryptococcal Study Group [8]. As expected, *C. neoformans* VNI was the most common genotype (76%) in HIV-infected people followed by *C. gattii* VGII (12.4%) isolates mostly from otherwise healthy patients [8]. The first two molecular types are also predominant in the environment (68.6 for VNI and 20.7% for VGII). Among the smaller numbers of veterinary cases, VGII is the most prevalent molecular type (73.7%). In Latin America, due to multilocus sequence typing analysis, the *C. neoformans* population is less diverse than that of the *C. gattii*.

These species are different regarding (a) pathogenicity, (b) prevalence among patients, (c) biochemical and physiological aspects, and (d) antifungal susceptibility testing results. It is fortunate that the MALDI-TOF mass spectrometry test is able to distinguish them. In the North American clinical setting, most genotyped *C. neoformans* belong to the VNI genotype as mentioned above (>90%) [5]. While pulmonary disease incidence is higher than other infections caused by these pathogens, the central nervous system disease caused by *C. gattii* is most frequent among AIDS patients [7]. Most cryptococcal clinical isolates in the USA are *C. neoformans*.

The issue of antifungal resistance is important in the clinical setting. Both CLSI and EUCAST have developed breakpoints (BPs) and epidemiological cutoffs (ECVs/ECOFFS) for certain species/antifungal combinations as discussed below. ECVs are available for the most common *Candida* and *Aspergillus* spp. and some commercial methods, but not for the cryptococcal isolates. BPs can categorize an isolate as either susceptible or resistant, while the ECV/ECOFFs will distinguish the wild type (WT, no known resistance mechanisms) from Non-WT (NWT, harboring resistant mechanisms).

### 1.2. Purpose Statement

The purpose of the present review was (1) to conduct a literature search regarding the available antifungal susceptibility methods for the *Cryptococcus* species; (2) to describe and provide available in vitro data by the various methods, especially those able to identify the non-susceptible or non-WT isolates; and (3) if possible, to advocate the potentially most useful method in the clinical setting.

## 2. Available Antifungal Agents

The antifungal agents available for the treatment of cryptococcal meningitis or other invasive infections are amphotericin B or a lipid formulation alone or combined with flucytosine followed by a consolidation phase with fluconazole, the latter being the choice agent for maintenance therapy [9,10,11]. In general, amphotericin B and the azoles (including isavuconazole, Astellas Pharma, Tokyo, Japan) show good in vitro activity against *Cryptococcus* spp. but these species are intrinsically resistant to the approved echinocandins [9,10,11,12,13,14,15]. The efficacy of isavuconazole was favorable compared to that of fluconazole for the treatment of murine cryptococcal meningitis, but this agent has not been approved for these infections [12,13,14,15]. The same applies to the new oral encochleate amphotericin B (reduced toxicity) that had a similar efficacy to parenteral amphotericin B plus flucytosine in a cryptococcal meningoencephalitis mouse model [16].

## 3. Antifungal Resistance Mechanisms for Treatment Agents

Antifungal resistance is common, especially among isolates infecting immunocompromised/AIDS patients and the incidence is mostly linked to prior drug exposure [11]. Triazoles and amphotericin B target the fungal cell by either direct attack and alteration of the synthesis of the enzyme Erg 11 or ergosterol depletion, respectively [11,17,18,19]. Resistance to flucytosine is generally due to the genetic mutations that impair the uptake of the agent or interfere with the target nucleic acid synthetic pathway [11]. In addition, the cell capsule formation may alter the cell wall (including melanin production) which also leads to therapy tolerance. In other cases, the formation of resistant and large titan cells has been reported (>10 μ) and was associated with stress adaptation/alteration [19]. Three efflux pumps are regulated independently by different transcription factors in response to fluconazole exposure. Deletion of *AFR1* in H99 and R265 drastically reduced the levels of resistance to the triazoles which indicated that *AFR1* is the major drug efflux pump [11,18]. However, the fluconazole susceptibility was not affected when *AFR2* or *MDR1* was deleted in both strains [18]. On the other hand, the target of the echinocandins is the glucan synthase Fks1, an important enzyme during cell wall synthesis. As mentioned above, the *Cryptococcus* spp. are inherently resistant to the echinocandins as cell changes lead to rapid or transient adaptation and resistance to these agents, including the new agent rezafungin [20]. Three publications included data from three new agents (rezafungin, manogepix, and VT-1598) versus *C. neoformans* [15,20]. As expected, MICs for the latter species were high for both the established echinocandins and rezafungin (2–≥8 µg/mL) and low for manogepix (0.03–2 µg/mL). The geometric means of VT-1598 were lower (0.016 and 0.039) than those for fluconazole versus *C. neoformans* (1.89) and *C. gattii* (2.71) [20].

## 4. Antifungal Susceptibility Methods for Testing Cryptoccal Isolates

Various antifungal susceptibility methods (reference and commercial) have been established for the detection of antifungal resistance which plays an important role in the clinical setting. The in vitro data could help to select the best treatment for a patient’s fungal infection and could identify the local or global antifungal resistance epidemiology. These methods, developed for the antifungal evaluation of yeasts species including the Cryptococcal isolates, are well known as summarized below.

## 5. Reference Methods for *C. neoformans* SC and *C. gattii* SC

The CLSI published its broth dilution method for yeasts in 1997, the M27A document [21,22]. Since then, this methodology has been revised to determine minimal inhibitory concentrations (MICs), including those for the cryptococcal isolates, and minimal effective concentrations (MECs) for the echinocandins vs. the molds [21]. The EUCAST also developed a broth microdilution method for testing the susceptibilities of yeasts and molds, as well as the cryptococcal species [21,23]. The differences between both reference methods are briefly summarized below as detailed by both groups.

## 6. Standard Testing Conditions for *Cryptococcus* Isolates

The CLSI and EUCAST recommendations for *Cryptococcus* isolates differ as follows: (round vs. flat microdilution trays; RPMI broth with 0.2% vs. 0.2% glucose, an inoculum size of 0.5–2.4 × 10^3^ vs. 0.5–2.4 × 10^5^, MICs determined after 72 h vs. 48 h, visual and spectrometric reading, amphotericin B endpoint: 100% vs. 90% [22,23] (EUCAST E. Def 7.3.2 2022). Despite these differences, the results obtained by both methods are supposed to be comparable. However, the problem is those differences could be important, because classification endpoints (BPs or ECVs/ECOFFs) are species and method dependent.

## 7. Yeast Nitrogen Broth

In addition to the reference RPMI broth, the yeast nitrogen base (YNB) broth, supplemented with 0.5% glucose and buffered to pH 7, was introduced to enhance the growth of *C. neoformans* and improve the MIC clinical relevance [24]. The MIC is determined by the spectrophotometer and defined as the lowest drug concentration that reduces 50% of the growth in the control well (drug-free). The inter-laboratory agreement of MICs by this method was excellent among three sites (83 and 96% agreement within 1 and 2 log dilutions, respectively) [25]. In a third study, 149 isolates of *C. neoformans* var. *neoformans* from Ugandan AIDS patients were tested using the RPMI and the YNB broths [26]. An overall agreement of 88% between the two microdilution methods was observed, but the MIC range using the YNB could be wider. The perception was that patients infected with strains with low MICs could be detected [26]. Most data are by the RPMI CLSI broth.

## 8. Antifungal Resistance Detectors: BPs and ECVs/ECOFFS

### Breakpoints and ECVs

The best predictors of clinical outcomes are the BPs. However, the development of BPs requires animal model pharmacokinetic/pharmacodynamic (PK/PD) data, ECVs, and, most importantly, the clinical/microbiological outcome data from clinical trials [27,28]. Although some EUCAST BPs have been developed mostly based on PK/PD data and MIC distributions, to the best of our knowledge CLSI BPs are not available for these species [27]. On the other hand, the ECV is a newer interpretive endpoint that identifies the NWT (mutants) strains [29]. The ECV development only requires in vitro data according to the guidelines in the M57 document as follows [29]: (1) defined by the iterative statistical method and (2) the modes of the distributions entering the pool must be at least one to two dilutions from the global/overall mode. This step ensures inter-laboratory agreement of MIC values by the same method [29,30,31,32]. Another requirement is that the BP or ECV should be based on the same methodology or the concept of a method-specific categorical endpoint [27,28,29,30,31]. It is interesting that Appendix B of the CLSI M59 document lists some yeasts as intrinsically resistant to the echinocandins, as follows: *C. krusei*, *C. lusitaniae*, and *Cryptococcus* spp. [30]. Furthermore, the wild-type MIC distributions, ECVs, and resistance mechanisms are needed for the establishment of BPs in addition to the correlation of in vitro vs. in vivo results from clinical trials [27]. It is not the ECV’s role to categorize a fungal isolate as susceptible or resistant as BPs do. The terms WT and NWT are not the same as “susceptible” and “resistant”.

## 9. Available Classification Endpoints for the *Cryptococcus* SC

Selected fluconazole CLSI ECVs for the *Cryptococcus* spp. and genotypes were developed according to the CLSI criteria listed in Table 1 and also in the M59S document, 4th ed. (Table 2 of the CLSI document, entitled ECVs for *Cryptococcus* spp. and basidiomycete yeasts) [32,33,34]. The CLSI ECV for *C. neoformans* non-typed isolates was defined as 16 μg/mL with both media. Therefore, isolates of this species for which the fluconazole MIC is 32 μg/mL (or >16 μg/mL as the ECV is 16 μg/mL) can be categorized as NWT or having resistance mechanisms [30,32,34]. The mode helps to evaluate the variability of the MIC data among different participant laboratories since at least three laboratories’ data should be analyzed (M57) [29,30,31,32].

The EUCAST has not proposed ECVs for any of the *Cryptococcus* spp. and fluconazole. However, ECVs for *C. neoformans* and amphotericin B (1 µg/mL) as well as posaconazole and voriconazole (0.5 µg/mL and 0.5 µg/mL, respectively) are available. In addition, EUCAST ECVs are available for *C. gattii* and amphotericin B (0.5 µg/mL) as well as for posaconazole (1 µg/mL). These ECVs are to be used when testing by the EUCAST E.Def 7.3, E.Def 9.4 and E.Def 11.0 Procedures (www.eucast.org, last accessed on 20 March 2023).

## 10. Incidence of Cryptococcal Infections as Reported from 2000 to 2016

Below, we have summarized fluconazole data reports from different areas in the world regarding the incidence of cryptococcal infections as published between 2000 and 2016 (Table 2). We have also documented MIC distributions from the collaborative study that defined triazole ECVs for Cryptococcal species for the CLSI M27 A method (Table 1 and Table 2) [30,34].

i. Aller et al. listed MIC data from Spain for 25 *C. neoformans* isolates, five of them being from therapeutic failure patients [35]. Fluconazole MICs were 0.5–≥16 μg/m by the CLSI method using the YNB broth, a final inoculum of 10^4^ CFU/mL, and 48 h of incubation. Some of these patients had prior oropharyngeal candidiasis and Cryptococcal antigen titers of >1:4000. Therapeutic failure was observed in five patients who were infected with isolates for which fluconazole MICs were ≥16 μg/mL (MICs > 16 could be classified as NWT or mutants). Four of these patients had previously had oropharyngeal candidiasis (OPC) and three had previous episodes of Cryptococcal infection; the five treatment failure patients had high Cryptococcal antigen titers in either serum or cerebrospinal fluid.
ii. In another study also using the CLSI method with the YNB broth, fluconazole MICs were: ≤8 µg/mL or WT value for >200 *C. neoformans* isolates [36]. These strains were recovered from 265 patients before fluconazole therapy for cryptococcal infection. A total of 11/116 patients relapsed, and fluconazole MICs increased from 1 µg/mL to 16–32 and 64 µg/mL. Given a CLSI ECV of 16 µg/mL for this method and medium, there were only 2/4 NWT strains in this set (MICs: 32 µg/mL and 64 µg/mL).
iii. Brandt et al. collected a total of 522 strains of *C. neoformans* isolates in the United Sates from 1992 to 1994 and 1996 to 1998 [37]. The CLSI fluconazole distribution was truncated (mode at the first drug concentration) and the number of NWT isolates (>16 µg/mL) was 16.
iv. Govender et al. reported CLSI data from a surveillance study in South Africa from 2003 to 2008 [38]. From the total of 482 *C. neoformans* isolates recovered, only 3 (0.6%) had CLSI fluconazole MICs of ≥16 μg/mL. Amphotericin B MICs and those of other triazoles were also low.

Two reports of EUCAST fluconazole MICs for *C. neoformans* were as follows:

v. Cordoba et al. summarized fluconazole MICs for 702 isolates, with a mode of 8 μg/mL [39]. As mentioned above, ECVs are not available by the EUCAST for this species/agent combination. Based on their own data, the authors calculated an ECV of 32 μg/mL and concluded that there were 16 non-WT isolates in their distribution [39].
vi. In the other publication by Perkins et al. [40], the “resistant endpoint” for fluconazole was ≥16 μg/mL; therefore, a total of 148/317 (48%) strains were classified as “resistant”; the mode was 16 μg/mL Although the distribution was reported, no EUCAST ECV data are available and hence there is no way to sort out the NWT (isolates harboring resistance mechanisms) among those 148 isolates [30,32].
vii. CLSI MIC data for 58 typed strains from Spain by Guinea et al. were found in the literature as follows: *C. neoformans* var. *grubii* (24; 42.9%), *C. neoformans* var. *neoformans* (11; 19.6%), the hybrid *C. neoformans* var. *grubii* × *C. neoformans* var. *neoformans* (19; 33.9%), and the co-existence of both *C. neoformans* var. *grubii* and the hybrid *C. neoformans* var. *grubii* × *C. neoformans* var. *neoformans* (2; 3.6% [41]. As shown in Table 1, MICs of 16 μg/mL (3.4%) were determined for two strains from patients with HIV infections; for those strains which are WT, the CLSI fluconazole MIC is equal to the ECV for this agent. These two WT isolates were categorized as “susceptible” to the new triazoles (MICs: 0.062 μg/mL) [41].
viii. Chen et al. documented CLSI fluconazole MICs for 89 isolates (48 from blood and 45 from CSF, 4 *C. gattii* isolates not in Table 2) [42]. The strains were identified/typed as *C. neofornans* var. *grubii* serotype A (89 isolates) and *C. gattii* serotype B (4 isolates). Of the 89 *C. neoformans* isolates, 30 (34%) were categorized as CLSI fluconazole-non-susceptible (MICs > 8 μg/mL) instead of NWT. It is difficult to know how many were 16 μg/mL or >16 μg/mL. Some of the “non-susceptible” isolates could be WT based on values equal to or below the ECV of 16 μg/mL.

Two studies reported Etest fluconazole data for small sets of *C. neoformans* isolates [43,44].

ix. In one study originating in Serbia, Etest fluconazole MICs of ≤8 μg/m were reported for almost half of the isolates 48.4%) [43]. So far, Etest ECVs are not available for the cryptococcal species.
x. In the other study, Etest fluconazole MICs were either <32 μg/mL (34 *C. neoformans*) and >32 μg/mL (3 isolates) [44]. These isolates were recovered from patients with cryptococcal meningitis in Yaoundé (Cameroon). Some comparisons between mostly CLSI and commercial methods will be discussed later.

As evident, the modes (highest number of isolates in each distribution) are either the same or 1–2 dilutions different using the same medium (either the reference RPMI [modes 2–4 µg/mL] or higher with the YNB broth [8 µg/mL]) [34]. This shows the influence of one of the testing conditions: the medium.

## 11. Commercial Methods: For Susceptibility Testing of *C. neoformans*/*C. gattii* SC Isolates

There are various commercial methods for antibacterial/antifungal susceptibly testing of isolates; we are summarizing data found for cyptococcal isolates in the literature by four of those methods: 1. the Sensititre YeastOne panel/plate (SYO, TREK Diagnostic Systems, Cleveland, OH, USA); 2. the Etest, bioMerieux Marcy-l’Etoile, France; 3. the automated VITEK^®^ 2, and 4. the flow cytometry method. Although commercial methods could be useful for the determination of MICs for fungal isolates, none of these methods, to our knowledge, have any means to categorize their MICs for the cryptococcal species. However, below, we summarize antifungal MICs obtained by these four procedures.

- Etest amphotericin B MICs were 2–4 μg/mL for three *C. neoformans* strains recovered from AIDS patients not responding to amphotericin B therapy or having reduced ergosterol content [45]. On the other hand, the MICs were 0.06–0.25 μg/mL for the nine strains isolated from patients responding to therapy [45]. The CLSI amphotericin B ECV is 2 μg/mL for *C. neoformans*, but not by the Etest. Defining amphotericin B ECVs for Etest and *C. neoformans* is warranted to better evaluate the utility of the Etest method for these species and agent with more isolates. However, this could be the best method for this agent as recommended by the CLSI for testing *Candida lusitaniae*.
- An early comparison of the CLSI method was conducted with the SYO assay for 20 isolates of *C. neofornans* among several *Candida* spp. [46]. Although the SYO method provided comparable data for most *Candida* spp., the agreement was 95% for amphotericin B within one dilution but much lower for fluconazole and 5FC (80% and 60%, respectively). The results were better when the comparison was within two dilutions [46]. The number of isolates evaluated was small, but the difference between the CLSI and SYO methods was 14%.
- In another study, MICs for 107 *C. neoformans* isolates were evaluated by the SYO colorimetric and CLSI methods [47]. The agreement within two dilutions with the CLSI M27 for amphotericin B, fluconazole, and flucytosine MICs was 76%, 98%, and 96%, respectively. Again, it appears the SYO and the M27 percentages of agreement are not satisfactory for testing amphotericin B. Therefore, neither method is recommended for this agent.
- CLSI, Etest, and SYO posaconazole MICs were compared for 15 isolates of *C. neoformans* [48]. The agreement with the reference method was better with the Etest than with the SYO method at 48 h, yielding 93 and 79%, respectively. Although other comparisons of posaconazole MICs had not yet been reported for this species at the time, the SYO was also found to be unsuitable for testing *C. neoformans* versus other antifungal agents [48].
- Ninety-two non-duplicate clinical and environmental *Cryptococcus* isolates were evaluated (57 *C. neoformans* and 35 *C. gattii* isolates) [49]. Isavuconazole Etest and the CLSI broth microdilution data were compared and no major discrepancies were observed (98%: >2-well dilution difference between these species and methods).
- CLSI, Etest, and VITEK^®^ 2. amphotericin B, fluconazole, flucytosine, and voriconazole MICs for 102 *C. neoformans* clinical isolates from South Africa were compared [50]. Fluconazole Etest MICs were similar to the reference data (95%) but not those of amphotericin B (83%). A ≥95% agreement was observed between VITEK^®^ 2 and CLSI data for fluconazole, flucytosine, and amphotericin B. Therefore, the VITEK^®^ 2 provided comparable MICs to those by the CLSI method in that study including those of amphotericin B; the results were also good for voriconazole (comparable MICs) (not in Table 3) [50]. However, more information is needed to cover other species.
- The newer flow cytometry method was assessed against the CLSI method to determine the in vitro antifungal susceptibility of 16 *C. neoformans* and 24 *C. gattii* strains to fluconazole [51]. MICs by the flow cytometry method were defined as the lowest drug concentration that showed ~50% of the count of acridine orange negative cells as compared to that of the growth control. According to their categorical classification, all *C. neoformans* isolates were “susceptible”. Applying the CLSI ECVs, all *C. neoformans* and 21 *C. gattii* could be WT; the three *C. gattii* isolates with higher MICs could be NWT. It is not clear which endpoints were used for their classification.

## 12. Conclusions

The available CLSI ECVs can categorize the most common Cryptococcal genotype isolates versus amphotericin B, flucytosine, and the triazoles. In addition, several commercial assays (e.g., the microdilution SYO, the agar diffusion Etest, the VITEK^®^ 2, and more recently the flow cytometry method) have been evaluated for testing some Cryptococcal isolates with satisfactory results. However, ECVs are not available for any of these commercial methods and species. It would be good if some of the commercial companies develop ECVs for their methods and thus more meaningful results would be obtained. ECVs for a variety of *Candida* are available and the same can be implemented for the Cryptococcal isolates. It is widely accepted that the reference methods are not the best or practical choice in most clinical laboratories. Although favorable equivalence results have been observed from the comparison of the commercial and CLSI methods, ECVs and BPs are species and method dependent. Unfortunately, the lack of suitable clinical data to establish BPs for commercial methods precludes that important step. In most instances, an important step is the identification of the isolates at the species level. However, the definition of ECVs for the commercial methods will allow a better interpretation (WT or non-WT) which, in the absence of BPs, will be more helpful in the clinical setting.

## Figures and Tables

**Table 1:** CLSI Fluconazole ECVs: *Cryptococcus neoformans*–*Cryptococcus gattii* SC obtained in 6 to 18 laboratories by the CLSI M27-A broth microdilution method.

Species/Genotype ^1^ MICs (No. of Labs)	Mode MIC ^2^	Statistical ECV ≥95% ^3^	Statistical ECV ≥97.5% ^3^
*C. neoformans* Non-typed isolates 4446 MICs (18 labs.)	4	16	16
VNI 1137 MICs (6 labs.)	2	8	8
*C. gattii* Non-typed isolates 137 MICs (6 labs.)	4	8	8
VGI 260 MICs (7 labs.)	4	8	16
VGII 101 MICs (7 labs.)	8	32	32

^1^ Data from >3 laboratories using the CLSI-RPMI broth [22]. ^2^ Mode, MIC most frequently obtained for each distribution. ^3^ Calculated epidemiological cutoff values (ECVs in µ/mL) comprising ≥95 or ≥97.5% of the statistically modeled population for which MIC distributions originated in at least three laboratories [30,31,34]. Mode: MIC most frequently obtained for each distribution.

**Table 2:** Cryptococcal incidence: method, medium, distributions, and non-WT endpoints ^1^. Fluconazole MIC data either as distributions or percentages of non-WT isolates.

Ref.	Method	No. of Isolates at Each MIC							Total	Date
		≤0.5	1	2	4	8	16	≥16		
[35] ^2^	CLSI (YNB)	**2**	4	2	**8**	4	1	4	25	2000
[36] ^2,3^	CLSI (YNB)					**260**	3	2	265	2009
[34] ^2,3^	CLSI (YNB)	19	23	73	84	**126**	26	8	359	2012
[37]	CLSI				**231**	189	85	17	522	2001
[38]	CLSI	27	82	**196**	138	30	9		482	2011
[34] ^4^	CLSI	483	543	1225	**1372**	569	180	74	4446	2012
[39]	EUCAST	17	19	43	116	**256**	172	79	702	2016
[40] ^5^	EUCAST						148/317 (48%)		317	2005
[41]	CLSI						2/58 (3.4%)		58	2010
[42] ^6^	CLSI						30/89 (34%)		89	2015
[34] ^7^	CLSI	19	39	69	**101**	29	3		260	2012
[43]	Etest					15/31 (48%)			31	2012
[44]	Etest						34	3	37	2015

^1^ CLSI/EUCAST/Etest: Fluconazole MICs determined by reference or commercial methods as reported/noted. ^2^ YNB: broth used in some studies, instead of the reference RPMI medium. Shaded: modal MIC or the highest number of MICs in each distribution. ^2,3^ *C. neoformans* non-typed isolates from the collaborative CLSI ECV definition study [34]. ^4^ Genotyped VGI isolates, from the collaborative CLSI ECV definition study [34]. ^5^ *C. neoformans*: Fluconazole MICs exhibited the lowest in vitro activity (48% at MIC ≥ 8 μg/mL) [40,43]. ^6^ *C. grubii*: 89 isolates serotype A and 4 isolates *C. gattii* (serotype B, (not in the Table) [42], a total of 89 isolates. ^7^ *C. gattii* VGI isolates distribution from the collaborative CLSI ECV definition study [34].

**Table 3:** Percentage agreement between reference and commercial susceptibility testing methods for *C. neorformans* and *C. gatii* isolates.

			Antifungal Agent and % Agreement ^1^					Ref.
Species	No. Isolates	Method	AMB	5FC	FLU	POS	ISA	
*C. neoformans*	3	Etest	2.0–4 ^2^					[45]
	9	Etest	0.06–0.25 ^2^					[45]
	20	SYO	95	60	80			[46]
	107	SYO	76	96	98			[47]
	15	Etest/SYO				93/79		[48]
	57	Etest					98	[49]
	102	Etest	83		95			[50] ^3^
		VITEK ^®^ 2	95	95	95			[50]
	16	Flow cytometry			16/16			[51]
*C. gattii*	35	Etest/SYO					98	[49]
	24	Flow cytometry			21/24			[51]

^1^ Agreement between the listed commercial method and CLSI M27-A method [22]. ^2^ Three isolates from patients not responding to amphotericin B therapy versus MICs from nine responders; MICs in µg/mL. SYO: Sensititre Yeast One. ^3^ The EA for voriconazole by the Etest was 91% [50].

## Data Availability

Not applicable.
